# Testosterone Deficiency, Cardiac Health, and Older Men

**DOI:** 10.1155/2014/143763

**Published:** 2014-04-10

**Authors:** G. Hackett, M. Kirby, A. J. Sinclair

**Affiliations:** ^1^Heartlands Hospital, Bordesley Green East, Birmingham, B9 5SS, UK; ^2^IDOP, University of Bedfordshire, Putteridge Bury Campus, Luton, Bedfordshire LU2 8LE, UK; ^3^The Prostate Centre, 32 Wimpole Street, London W1G 8GT, UK; ^4^The Luton & Dunstable Hospital, Lewsey Road, Luton, Bedfordshire LU4 0DZ, UK

## Abstract

Low levels of testosterone are manifested by erectile dysfunction, reduced sexual desire, and loss of morning erections with increasing numbers of men are being diagnosed and require treatment. The prevalence rates of testosterone deficiency vary according to different studies but may be as high as 40% in populations of patients with type 2 diabetes. There is increasing evidence that testosterone deficiency is associated with increased cardiovascular and all-cause mortality. Screening for low testosterone is recommended in a number of high risk groups including those with type 2 diabetes and metabolic syndrome. There are recent data to suggest that testosterone replacement therapy may reduce cardiovascular mortality as well as improving multiple surrogate markers for cardiovascular events. Specific clinical trials of testosterone replacement therapy are needed in selected populations but in the meantime we must treat patients based on the best current evidence.

## 1. Introduction

The current ISSAM (International Society for Study of the Aging Male), EAU (European Association of Urology), and BSSM (British Society for Sexual Medicine Association) definition of Late Onset Hypogonadism [1, 2] is

*“A biochemical syndrome associated with advancing age and characterised by a deficiency in serum androgen levels with or without a decreased genomic sensitivity to androgens. It may result in significant alterations in the quality of life and adversely affect the function of multiple organ systems”. *

This state of hypogonadism causes a global decrease in energy and a decrease in the feeling of well-being. It also causes a change in sexual function and has other endocrine and metabolic repercussions. These can affect bones, muscles, and lipids, as well as cognitive function Testosterone Deficiency Syndrome (TDS) or Late Onset Hypogonadism which is defined on the basis of clinical symptoms associated with abnormal testosterone levels. The European Male Aging Study [3] (EMAS) studied 3369 men aged 40−79 at 8 European centres and concluded that the 3 following cardinal symptoms were most likely to be related to low levels of testosterone.

*Erectile Dysfunction, Reduced Sexual Desire, and Loss of Morning Erections.* Other symptoms such as hot flushes, sweats and tiredness, loss of vitality, reduced shaving frequency, gynaecomastia, depressed mood, poor concentration, and sleep disturbance were regarded as less specific.

Recent guidelines suggest that a level of total testosterone of <8 nmol/L or free testosterone of less than 180 pmol/L requires testosterone replacement therapy and total testosterone of >12 nmol/L or free testosterone of >225 pmol/L does not. Between these levels a trial of therapy for a minimum of 6 months should be considered based on symptoms [1, 2]

## 2. Biochemical Assessment of Hypogonadism

Total testosterone should be measured between the hours of 7 and 11 am on 2 occasions at least 1 month apart and ideally be assessed by mass spectrometry (ID-GCMS). Equilibrium dialysis is currently the gold standard for free testosterone as immunoassays based on analogue displacement are currently inaccurate [1, 2, 4].

The Endocrine society [4] recommends that men with the following conditions should be screened for low testosterone routinely:

- type 2 diabetes,
- metabolic syndrome,
- moderate to severe chronic lung disease,
- osteoporosis,
- HIV,
- history of infertility,
- treatment with steroids, opiates (even medically prescribed), and anticonvulsants,
- alcohol abuse.

## 3. Low Testosterone and Increased Mortality

There is increasing evidence from multiple long-term studies that TDS is associated with increased cardiovascular and all-cause mortality [5–13]. Two recent meta-analyses have looked at a large number of long-term studies linking low testosterone to increased cardiovascular and all-cause mortality [14, 15]. Araujo et al. [14] concluded that the evidence for a link between low testosterone and increased mortality was strong but concluded that most studies involved issues in cohort selection and choice. They concluded that a decrease of 2.1 standard deviations in total testosterone was associated with a 25% increase in mortality. Haring [15] et al. looked at the data in terms of several models and found that even after strict adjustment for comorbidities there was a consistent link between mortality risk and testosterone level throughout the studies but that this did not prove causation (Table 1).

The EMAS group [16] recently reported 4.3 year follow-up data on 2599 men aged 40–79 and concluded that men with a baseline TT of 8 nmol/L or less and sexual symptoms had a 3-fold increased mortality and a 5-fold increased risk of cancer death. There authors concluded that there is a small number of men with low testosterone at considerable risk of early death.

A recent 10 year study from Western Australia involving 3690 men followed up from 2001–2010 concluded that TT and FT levels in the normal range were associated with decreased all-cause and cardiovascular mortality, for the first time suggesting that both low and DHT are associated with all-cause mortality and higher levels of DHT reduced cardiovascular risk [17].

Six published studies usually involving small samples have shown that low TT and FT are associated with CAD and 4 have shown no association [18]. Four studies have shown inverse associations between low TT or FT (Table 2) and the severity of CAD [18]. One involved 803 men assessed by Gensini score, based on the location and number of stenotic coronary artery segments and degree of luminal narrowing [19]. Such studies do not establish whether low TT or FT is a cause or a consequence of CAD.

### 3.1. Mortality Studies from High Risk Groups

Malkin et al. [20] followed up 930 men referred with coronary artery disease for 6.9 years. The prevalence of hypogonadism was 24%, mortality rates were 21% versus 12% (*P* = 0.002) for hypogonadal men versus eugonadal, and the study was halted early at 6.9 years. Only beta-blocker therapy and left ventricular failure were found to have a greater influence on survival.

Muraleedaran [21] et al. screened a primary care diabetic population of 587 patients and followed them up for 5.8 years. They found that 475 of men had normal TT levels, 22% were overtly hypogonadal (<8 nmol/L), and 31% were in the borderline range. These percentages were in close agreement with earlier publications by Kapoor [22] et al. and Hackett et al. [23]. The mortality rate [21] over 5 years in the hypogonadal group was 17.2% versus 9% in the normal testosterone cohort. The effect of treatment with TRT reduced the mortality rate of treated cohort (8.4%) to that of the eugonadal group whereas the mortality for the untreated remained high at 19.2%, after adjustment for all confounding factors.

### 3.2. Effect of Low Testosterone on Surrogate Markers for Cardiovascular Risk

Decreases in serum total cholesterol (TC) have been noted as early as after 4 weeks [23] but most studies have reported a decrease after 3 months [24]. Greater reductions were seen in obese men [24] with metabolic syndrome. The MRFIT [25] study showed that hypogonadal men had slightly increased triglycerides and HDL, leading to the suggestion that TRT might be expected to lower triglycerides and HDL. A recently published 5-year registry involving 230 men treated with long acting testosterone showed highly significant reductions in TC, LDL, and triglycerides with increase in HDL, associated with significant reduction in weight, BMI, and visceral fat [26].

The decrease in serum triglycerides follows a similar pattern: after 4 weeks with decrease over 9 months [25] and maximum effect at 12 months [25]. The decrease in low-density lipoprotein cholesterol seems somewhat slower: after 3 months, after 40–44 weeks, or after 12 months [25]

Studies have found both an increase and decrease in HDL cholesterol [26–28] dependent on the presence of diabetes or the use of statins. TRT has also been shown to reduce fibrinogen to levels similar to fibrates. Low androgen levels are associated with an increase in inflammatory markers [24]. A decline was noted in IL6 and TNF-alpha within 16 weeks [27] and in another study after 16 weeks [24]. In the Moscow study, C-reactive protein was reduced by TRT at 30 weeks versus placebo [27].

Several of the above studies have shown reduction in waist circumference, visceral fat, and BMI [26–28]. Preliminary longer term studies suggest that considerable weight loss can be seen for up to 4 years. Placebo controlled studies in untreated hypertension are difficult to conduct for ethical reasons. In some studies, a decline in diastolic blood pressure has been observed, after 3–9 months [24, 26] and in systolic blood pressure [24, 26]. Maximum effects were observed after 12 months and up to 5 years [24, 26].

### 3.3. Effects on Angina Threshold and Heart Failure

Men with angiographically proven CAD (coronary artery disease) have significantly lower testosterone levels [29] compared to controls (*P* < 0.01) and there was a significant inverse relationship between the degree of CAD and TT (total testosterone) levels (*r* = −0.52, *P* < 0.01). Nearly 25% of men presenting for coronary angiography had testosterone levels in the low hypogonadal range and some 50% had a TT of less than 11 nmol/L [30].

Studies have shown pharmacological doses of testosterone to relax coronary arteries when injected intraluminally [39] and to produce modest but consistent improvement in exercise-induced angina and reverse associated ECG changes [40]. The mechanism of action is via blockade of calcium channels with effect of similar magnitude to nifedipine [40].

In men with chronic stable angina pectoris, the ischaemic threshold increased after 4 weeks of TRT and a recent study demonstrates improvement continuing beyond 12 months [39, 40]. Exercise capacity in men with chronic heart failure increased after 12 weeks [30], predominantly through the improvement in skeletal muscle performance.

Lower levels of endogenous testosterone have been shown to be associated with longer duration of the QTc interval and TRT has been shown to reverse this effect [41]. Carotid artery intimal thickness is associated with low endogenous testosterone suggesting an increased risk of atherosclerosis [31].

A trial of 209 elderly frail men [32] over 65 randomised to receive either placebo or 100 g of topical testosterone gel was terminated early as there were 23 cardiac events (2 deaths) in the 106 men in the testosterone group versus 5 in the placebo group, despite positive results in study end points. These events included myocardial infarction and dysrhythmias and hypertension. The authors conceded that there were more cardiovascular comorbidities in the active treatment group and that the starting dose and escalation were outside the product licence. The active treatment group had more severe CAD. The study involved rapid escalation up to 150 mg per day, above the manufacturers recommended dose and many of the events were reported with inadequate validation.

### 3.4. Testosterone, Insulin Resistance, and Type 2 Diabetes

Studies have shown an inverse relationship between serum testosterone and fasting blood glucose and insulin levels [33]. Both hyperinsulinaemia and low testosterone have been shown to predict the development of type 2 diabetes (T2D) [42, 43]. Medications such as chronic analgesics, anticonvulsants, 5ARIs, and androgen ablation therapy are associated with increased risk of testosterone deficiency and insulin resistance [1, 2].

Hypogonadism is a common feature of the metabolic syndrome [42, 43]. Intraabdominal adiposity (IAA) drives the progression of multiple risk factors directly, through the secretion of excess free fatty acids, inflammatory adipokines, and decreased secretion of adiponectin [44]. The important contributions of IAA to dyslipidaemia and insulin resistance provide an indirect, though clinically important, link to the genesis and progression of atherosclerosis and cardiovascular disease [44]. The presence of excess IAA is an important determinant of cardiometabolic risk. The INTERHEART study [45] involving 29,972 participants examined the contributory factors involved in first AMI (acute myocardial infarction) and found IAA to be an important predictive factor and recommended waist circumference or hip-waist ratio as a standard measurement of cardiovascular risk. Women with T2D or metabolic syndrome characteristically have low SHBG and high free testosterone [6]. The precise interaction between insulin resistance, visceral adiposity, and hypogonadism is, as yet, unclear but the important mechanisms are through increased aromatase production, raised leptin levels, and increase in inflammatory kinins [46].

In obese males, levels of testosterone are reduced in proportion to degree of obesity. The first step in reducing visceral fat is diet and lifestyle change [46]. Patients should be advised to switch to a low glycaemic diet, providing carbohydrate that does not increase glucose levels that means reducing potatoes and bread and substituting natural rice and full corn. Men should be encouraged to combine aerobic exercise with strength training. As muscle increases, glucose will be burned more efficiently and insulin levels will fall. A minimum of 30 minutes exercise three times weekly should be advised [46].

Men with low testosterone levels show less diurnal variation compared with younger men with normal levels [1]. Testosterone increases levels of fast-twitch muscle fibres [46]. By increasing testosterone, levels of type 2 fibres increase and glucose burning improves. Weight loss will increase levels of testosterone and augment the effects of lifestyle and exercise advice [47].

Diabetes specialists have traditionally considered the fall in testosterone level as being a consequence of obesity but studies now clearly show that low testosterone leads to visceral obesity and metabolic syndrome and is also a consequence of obesity [4]. Large long-term studies have shown that baseline levels of testosterone predict the later development of type 2 diabetes [25, 42, 43]. In the case of MMAS [43], a baseline total testosterone of less than 10.4 nmol/L was associated with a greater than 4-fold incidence of type 2 diabetes over the next 9 years and NHANES-III followed up men from as young as 20 and found a similar 4 times greater prevalence independent of obesity or ethnicity [42]. Diabetes UK data 2010 [48] showed the prevalence of type 2 diabetes in men aged 35–44 to be doubled that of women despite men in that age group having lower levels of obesity and taking more exercise. This effect was even more marked in southern Asian men [48]. A study has recently commenced in Australia to establish whether treating young obese men with low testosterone will reduce the incidence of type 2 diabetes.

There is high level evidence that TRT improves insulin resistance, as measured by HOMA-IR [49–52], and reduces HbA1c [49] by approximately 0.7% by 18 months [53] and inflammatory markers (CRP, IL6, and TNF-alpha) in men with type 2 diabetes and metabolic syndrome [26–28]. There is also high level of evidence for reduction in total cholesterol, weight, BMI [26–28], and visceral fat (a significant marker for CV risk) and improvement of lean muscle mass. The BLAST study [53, 54] suggested that men with depression (23% of the cohort with diabetes) were markedly less responsive to testosterone and that improvement in metabolic parameters required sustained levels of testosterone above 12 nmol/L [53, 54] (Table 3).

A recent 5 year registry of 255 men age 36–69 [26] treated with long acting TU has shown mean reductions in waist circumference of 8.5 cm, weight reduction of 15.5 kg, total cholesterol by 2.4 mmol/L, reduction in HDL, and triglycerides with HbA1c by 0.9% (7.06 to 6.16).

### 3.5. The Effects of Testosterone Replacement Therapy on Cardiovascular Mortality

**Figure 1:**
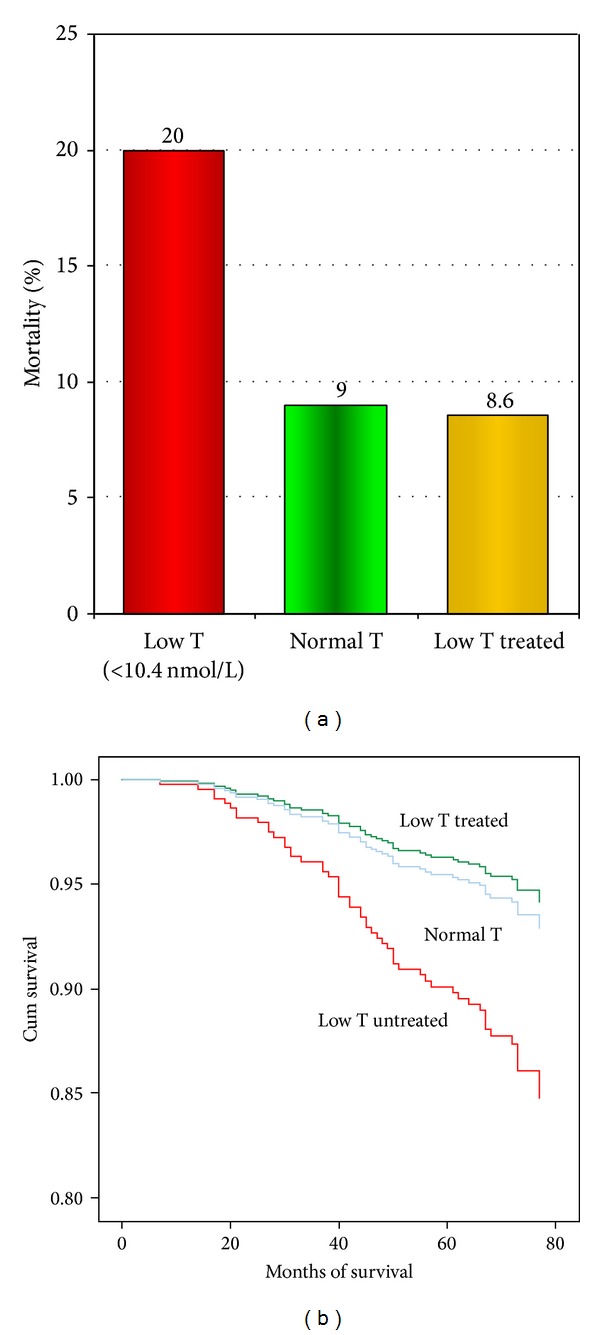
Low testosterone predicts increased mortality and testosterone therapy improves survival in 587 men with type 2 diabetes (mean follow-up: 5.8 years) Muraleedaran et al. [21].

A prospective recent study of 587 men with type 2 diabetes [21] involved 5.8 years follow-up. Low testosterone was defined as TT <10.4 nmol/L. Fifty-eight men were treated with testosterone for 2 years or more. The mortality rate was 20% in the untreated group and 9.1% in the normal group independent of comorbidities and therapies. Mortality was 8.6% in the treated group (*P* = 0.049) (Figure 1).

A similar retrospective US study involved 1031 men with 372 on TRT. The cumulative mortality was 21% in the untreated group versus 10% (*P* = 0.001) in the treated group with the greatest effect in younger men and those with type 2 diabetes [55]. In a recent paper of 145 patients with first ischaemic stroke and diabetes, 66% were found to be hypogonadal, and in the testosterone treated group 7% had a recurrence of stroke in 2 years versus 16.6% in the control group with 28% of the treated men returning to work versus 6% of the control group. There were significant improvements in lipid profile and HbA1c [56].

A recent retrospective US study of 8709 men [57] with baseline TT of 10.4 nmol undergoing coronary angiography involved follow-up for mean 840 days. In the cohort of 7486 patients not receiving testosterone therapy, 681 died, 420 had MIs, and 486 had strokes. Among 1223 patients receiving testosterone therapy, 67 died, 23 had MIs, and 33 had strokes. At first sight these results would closely agree with the findings in [21, 55], but a complex statistical analysis reversed the trend and concluded that there was a greater risk in the TRT group. There were concerns that 1132 patients experiencing events were excluded because they were prescribed TRT after the event when surely these should have been included in the untreated group, increasing the events by 70%.

The baseline TT was 1 nmol/L lower in the treated group and previous studies show that this may increase the mortality by up to 30%, yet this was not considered in the 50 confounders for analysis. Symptoms were not considered, yet these are key to the diagnosis of hypogonadism. Men were likely to be treated with TRT on the basis of symptoms if they suffered from erectile dysfunction, and the presence of ED has been shown to be an independent risk factor, particularly in hypogonadal men, increasing the risk of cardiac events by over 50% [16, 58].

Therapies used were mainly short acting injection and patches, both with high discontinuation rates. 16% filled only one prescription but were included in the “treatment” group. Only 60% had any record of a follow-up testosterone level and in those the mean treatment level was 10.2 nmol/L, suggesting suboptimal therapy.

Despite these issues, the paper was given an editorial and a separate paper warning patients about the risks of testosterone was included in the same journal [59]. The level of criticism of this paper led to an immediate revision of the conclusions shortly after publication. A recent online publication on ischaemic heart disease mortality in men concluded optimal androgen levels are a biomarker for survival [17]. A recent systematic and meta-analysis of placebo-controlled trials of T therapy lasting more than 12 weeks concluded that testosterone therapy may increase the risk of cardiovascular-related events [60] but most studies involved small cohorts with a small number of events but once again the nonrandomised studies failed to consider the impact of symptoms as an indication for TRT prescribing.

A meta-analysis of 1000 patient years [61] versus placebo suggests a slight reduction in myocardial infarction and CVA but a reduction in coronary interventions. There was a 6% incidence of raised haematocrit (>50%) without significant consequences and no deaths in the active treatment group versus 5 in the placebo cohort.

## 4. Long-Term Safety of Testosterone Therapy on the Prostate

At least 7 observational studies have reported no association of LUTS with serum testosterone level and 5 have shown an inverse relationship [62]. No studies to date show an increase in LUTS/BPH symptoms with higher serum testosterone levels [62]. A recent long-term registry of 5 years TRT shows sustained reduction in IPSS, postresidual volume, and bladder wall thickness, despite minor increase in prostate volume [63]. Another 5-year study involving a smaller cohort showed no impact on LUTS/BPH parameters [64]. As TRT has been shown to upregulate PDE5 [65] and enhance the effect of PDE5Is (now an accepted therapy for both ED and LUTS), it no longer seems logical to advice avoidance of TRT in men with mild to moderate BPH.

Calof et al. [61] also found that patients on testosterone were 12 times more likely to get a prostate biopsy but no more likely to have a positive finding. Several meta-analyses have failed to show a link between TRT and development of prostate cancer [66] but some studies have shown a tendency for more aggressive prostate cancer in men with low testosterone. One recent study of 279 consecutive patients referred for biopsy on the basis of abnormal DRE or raised PSA found that* low* bioavailable testosterone and high SHBG were associated with a 4.9- and 3.2-fold risk of positive biopsy [67].

Current EAU, ISSAM, and BSSM guidance [1, 2] is that there is “no evidence TRT is associated with increased risk of prostate cancer or activation of subclinical cancer.”

Despite these conclusions, many patients are deprived of clinical and metabolic benefit because of minor physiological increases in PSA and concerns that no long-term study has conclusively proved absolute safety.

## 5. Effects of Androgen Ablation Therapy

Men with prostate cancer, treated with androgen deprivation, develop an increase of fat mass with an altered lipid profile. Total cholesterol by 9, 7, 11, and 26.5%, respectively. These patients also appear to develop insulin resistance, hyperinsulinemia, and hyperglycaemia. The risks of diabetes mellitus increase by 44% and mortality of cardiovascular diseases by 16% during a follow-up of up to 10 years [68]. The authors concluded that before commencing ADT, the overall health, comorbidities, and life expectancy of the patient need to be fully assessed.

## 6. Testosterone and Erectile Dysfunction

Several studies have clearly shown that TRT can be effective as monotherapy for ED [26, 28], in men without other multiple comorbidities. A recent double blind placebo controlled study in men with diabetes showed prompt improvement in IIEF in men with severe hypogonadism and a secondary improvement with 12 to 18 months of therapy, dependent on obtaining prolonged sustained levels in the normal range [69]. Results from a 5-year registry showed 12.3-point improvements in IIEF in 260 men treated with long acting TU [64]. A systematic review and meta-analysis of placebo-controlled studies published in the past 30 years aimed to study effects of testosterone on the different domains of sexual life. This latter study concluded that T treatment might be useful for improving vasculogenic ED in selected subjects with low or low-normal T levels [70].

Erectile dysfunction is an established marker for future cardiovascular risk and the major presenting symptom leading to a diagnosis of low testosterone [58]. Current guidelines suggest that* all* patients presenting with ED, irrespective of age, should be screened for low testosterone, as it is a* potentially curable cause of ED*, especially in men without othercomorbidities [1, 2]. NICE guidance [71] suggests that all men with type 2 diabetes be assessed for ED* annually *and the GP contract now includes routine assessment for ED, and increased demand for testosterone supplementation will be a natural consequence of this correction of previous underdiagnosis and undertreatment [72].

## 7. Conclusions

Men with low testosterone usually present with bothersome symptoms, particularly ED, and require treatment to address those problems, not simply for cardiovascular prevention purposes. The benefits of conventional cardiovascular risk reduction with exercise and weight reduction are fundamental to management but are frequently unsuccessful. There is a considerable body of evidence that low testosterone is associated with increased cardiovascular and cancer mortality. A policy of taking little or no action for these men based on concerns of increased cardiovascular and cancer risk associated with physiological replacement would seem illogical. There is considerable evidence of modest cardiac and metabolic benefits that are shown to reduce cardiovascular risk plus sexual, mood, and quality of life changes associated with restoring testosterone levels. These may add up to substantial benefit to many patients. These benefits may potentially denied to patients by fears over prostate and cardiac risk that is not currently supported by evidence. Ideally, we need large long-term studies to resolve these issues with certainty but such studies are unlikely to be done for logistic and financial reasons. Until then patients require advice and treatment based on the current best evidence.

## Figures and Tables

**Table 1:** Association of low testosterone levels with all-cause mortality by different cut-offs from recent studies.

Cut-off for the definition of low total testosterone (TT)	MMAS; [8] TT < 6.94 nmol/L (200 ng/dL)	Wang; [31] TT < 8.0 nmol/L (230 ng/dL)	Rancho Bernardo; [7] TT < 8.36 nmol/L (241 ng/dL)	Male veterans study; [32] TT < 8.7 nmol/L (250 ng/dL)	HIM; [33] TT < 10.41 nmol/L (300 ng/dL)	EPIC; [6] TT < 12.5 nmol/L (360 ng/dL)	Age-specific cut-off <10th percentile
Low TT (*n*)	34	69	82	98	241	474	
Model 1	1.59 (0.83; 4.02)	1.96 (0.93; 3.63)	2.21 (1.26; 3.89)**	2.24 (1.41; 3.57)**	1.33 (0.93; 1.90)	1.28 (0.95; 1.72)	2.21 (1.40; 3.49)**
Model 2	2.12 (1.01; 4.46)*	2.08 (1.12; 3.86)*	2.33 (1.33; 4.12)**	2.10 (1.34; 3.29)**	1.28 (0.89; 1.84)	1.20 (0.88; 1.62)	2.26 (1.43; 3.59)**
Model 3	2.50 (1.18; 5.27)*	2.24 (1.21; 4.17)*	2.53 (1.43; 4.47)**	2.32 (1.38; 3.89)**	1.37 (0.95; 1.99)	1.28 (1.93; 1.75)	2.35 (1.47; 3.74)***
Model 4	2.68 (1.19; 6.04)*	2.13 (1.06; 4.26)*	2.56 (1.38; 4.76)**	1.92 (1.18; 3.14)**	1.11 (0.72; 1.69)	1.10 (0.78; 1.56)	2.25 (1.35; 3.75)**

Model 1: adjusted for age. Model 2: adjusted for age, and WC. Model 3: adjusted for model 2, smoking (3 categories), high-risk alcohol use, and physical activity. Model 4: adjusted for model 3, renal insufficiency, and DHEAS. HR: hazard ratio; CI: 95% confidence interval; CVD: cardiovascular disease; WC: waist circumference; DHEAS: dehydroepiandrosterone sulfate.

*P < 0.05.

**P < 0.01.

***P < 0.001.

**Table 2:** Association between testosterone level and severity of coronary artery disease.

Study name	Subfraction of testosterone used for analysis	Method of measuring CAD severity	Main findings	Remarks
Dobrzycki et al. [34] (CCS, *n* = 96)	TT, FT, FAI	Duke index*	Inverse correlation between FT and CAD severity	*r* = −0.69, *P* = 0.048
Rosano et al. [35] (CCS, *n* = 129)	TT	Coronary artery score**	Inverse correlation between TT and CAD severity	*r* = −0.52, *P* < 0.01
Li et al. [36] (CCS, *n* = 803)	TT	Genisi score***	Inverse correlation between TT and CAD severity	*r* = −0.188, *P* < 0.001
Phillips et al. [37] (CCS, *n* = 55)	TT, FT	Visual estimation of coronary artery occlusion and calculation of mean percent occlusion****	Inverse correlation between TT and FT levels and CAD severity	TT: *r* = −0.43, *P* < 0.02; FT: *r* = −0.62, *P* < 0.001

CAD indicates coronary artery disease; CCS: case-control study; FAI: free androgen index; FT: free testosterone; TT: total testosterone.

*Duke prognostic coronary artery index: a prognostic tool involving the extent and severity of atherosclerotic lesions in coronary arteries.

**Coronary artery score: authors multiplied the degree of coronary artery obstruction by the number of stenoses.

***Genisi score: calculated based on location and number of stenotic coronary artery segments, and degree of luminal narrowing.

****Authors visually estimated the maximum percent reduction in luminal diameter of the left main, left anterior descending, left circumflex, and right coronary arteries. The mean of these 4 values was used to estimate CAD severity.

**Table 3:** Outcome of therapy with long acting TU in a population of men with type 2 diabetes and hypogonadism (BLAST) Hackett et al. IJCP Dec 2013 [38].

	HbA1c (%) >7.5	Weight (kg)	BMI (kg/m^2^)	WC (cm)	TC (mmol/L)	EF (TT < 8 nmol/L)	AMS (points)	HADS-D	GEQ (% imp)
30 weeks	−0.41	−0.7	−0.3	−2.5	−0.25	+3.0	−5.3	−1.01	46
*P* value	**0.007 **	0.13	**0.01 **	**0.012 **	**0.025 **	**0.006 **	0.095	0.64	**<0.001 **
82 weeks	−0.89	−2.7	−1.00	−4.2	−0.19	+4.31 +9.57 PDE5I	−8.1	−2.18	67–70
*P* value	**0.009 **	**0.016 **	**0.019 **	**<0.001 **	**0.035 **	**0.003 **	**0.001 **	**0.001 **	**0.0001 **
